# First case of successful eradication of the sweet potato weevil, *Cylas formicarius* (Fabricius), using the sterile insect technique

**DOI:** 10.1371/journal.pone.0267728

**Published:** 2022-05-12

**Authors:** Chihiro Himuro, Tsuguo Kohama, Takashi Matsuyama, Yasutsune Sadoyama, Futoshi Kawamura, Atsushi Honma, Yusuke Ikegawa, Dai Haraguchi

**Affiliations:** 1 Okinawa Prefectural Plant Protection Center, Naha, Okinawa, Japan; 2 Ryukyu Sankei Co., Ltd, Naha, Okinawa, Japan; 3 Faculty of Agriculture, University of Ryukyus, Nishihara, Okinawa, Japan; University of Thessaly School of Agricultural Sciences, GREECE

## Abstract

This paper presents the first case of the successful eradication of a Coleoptera pest species over a wide area using a combination of male annihilation technique (MAT) and sterile insect technique (SIT) application. The sweet potato weevil, *Cylas formicarius*, is one of the most destructive and widely distributed pests of sweet potato, *Ipomoea batatas*. A project to eradicate it was launched in 1994 on Kume Island, Okinawa Prefecture, Japan. The MAT application was first used from November 1994 to January 1999 to reduce the density of wild populations. The distribution and densities of weevils were assessed by trapping them and surveying infestation rates in wild hosts and sweet potatoes in the field. The *C*. *formicarius* populations were suppressed by approximately 90% and plant infestations were reduced from 9.5% to less than 0.1% by using the MAT. Then, hundreds of thousands to millions of sterile weevils were released each week (ca. 460 million in total from 1999 to 2012). As a result, based on an analysis of 12748 stems and 48749 tubers, no weevil infections were detected in the stems or tubers of sweet potato since 1997. Since 2009, almost no wild weevils were captured in traps, and in wild host and sweet potato surveys no weevils have been found in any of the 580 locations and 8833 samples since October 2011. As of 28 December, 2012, *C*. *formicarius* is considered to have been eradicated from Kume Island. This paper describes the process of eradicating *C*. *formicarius* using SIT application integrated with MAT application for the first time and discusses some of the main challenges associated with the weevil eradication campaignl.

## Introduction

The sterile insect technique (SIT) is an environmentally friendly pest control technique that is applied in the integrated, area-wide control of major pests. It can be used to suppress, contain, or eradiate introduced and native populations and to prevent establishment or reestablishment after eradication. It was first applied on a large scale to the New World screwworm *Cochliomyia hominivorax* (Coquerel) [1, 2], which was successfully eradicated from the southern states of the USA, Mexico, Central America and most of Panama [3, 4]. Due to its success, it was subsequently applied to a number of other insect pest species [5]. The principle of the SIT is that the target pests are reared in large numbers (mass-reared), reproductively sterilized, and then released into the wild. When sterile males mate with wild females, they do not produce viable offspring. The constant release of sterile insects on an area-wide basis results in an increasingly rapid decline in the overall population over several generations [2]. This technique has been successfully used against a number of insect pest species, including melon fly *Bactrocera cucurbitae* (Coquilett), Mediterranean fruit fly *Ceratitis capitata* (Wiedemann), pink bollworm *Pectinophora gossypiella* (Saunders), codling moth *Cydia pomonella* (L.), and tsetse fly *Glossina austeni* (Newstead) [6, 7]. In Coleoptera species, area-wide eradication is generally achieved through the use of pheromones and insecticides [8–10]. Although eradication of white grub cockchafer (*Melolontha vulgaris* F.) [11] using SIT application in an experimental field has been attempted, there is yet to be an area-wide integrated pest management (AW-IPM) program that integrates the SIT against any Coleoptera in the field [7].

The sweet potato weevil, *Cylas formicarius* (Fabricus), is one of the most destructive and widely distributed pests of sweet potato, *Ipomoea batatas* (L.) Lam. in the South Pacific, Caribbean Basin, and some Central and South American countries [12–15]. Infested roots induce the production of terpenoids, which may make even slightly damaged roots inedible to humans and animals [16, 17]. Therefore, even low weevil densities can cause high crop losses and prevent trade. However, losses often reaching 60–100% [13]. *Cylas formicarius* was first found in Japan in 1903 [18] and its distribution has since then expanded throughout the southwestern islands of Japan [14, 19]. In Japan, *Ipomoea* spp. are their main host plants [20]. In a survey conducted in 1988 in cultivation fields in Naha City and Yomitan village, Okinawa, Japan, the infection rate in the stems and tubers of sweet potato ranged from 20% to 100% [70]. Some of their wild host plants include blue morning glory *Ipomoea indica* (Burm.) Merr. and railroad vine *Ipomoea pes-caprae* (L.) Sweet. The movement of the weevil and its host plants, including sweet potato, from infested to noninfested areas, is strictly regulated under the Japanese Plant Protection Act. The eradication of weevils in the southwestern islands is therefore an important issue in the field of agriculture and trade.

This work presents the first attempt at using SIT application to eradicate *C*. *formicarius* over a wide area. To this end, we have developed methods for mass-rearing, sterilization, marking, release, and evaluation of pest control effectiveness. The successful application of SIT requires information of the ecology of the target population, including estimates of the absolute density of the adult population, and how that density changes over time (population dynamics) [21–24]. We conducted a distribution survey and population estimation during the pre-project. The SIT application is one of the few control techniques that is inverse density-dependent and is most efficient at low pest densities [23]. Therefore, the SIT would best be applied when the pest density is low due to prior attempts at pest population reduction, such as chemical insecticides, host removal, mass-trapping, and male annihilation. Integrating the SIT application as a final component into an AW-IPM program ultimately convert the treatment area into a pest-free area [25, 26]. In this paper, we report on the first successful eradication of *C*. *formicarius* using SIT application over a wide area and discuss some of the challenges faced.

## Materials and methods

### Target area

**Fig 1:**
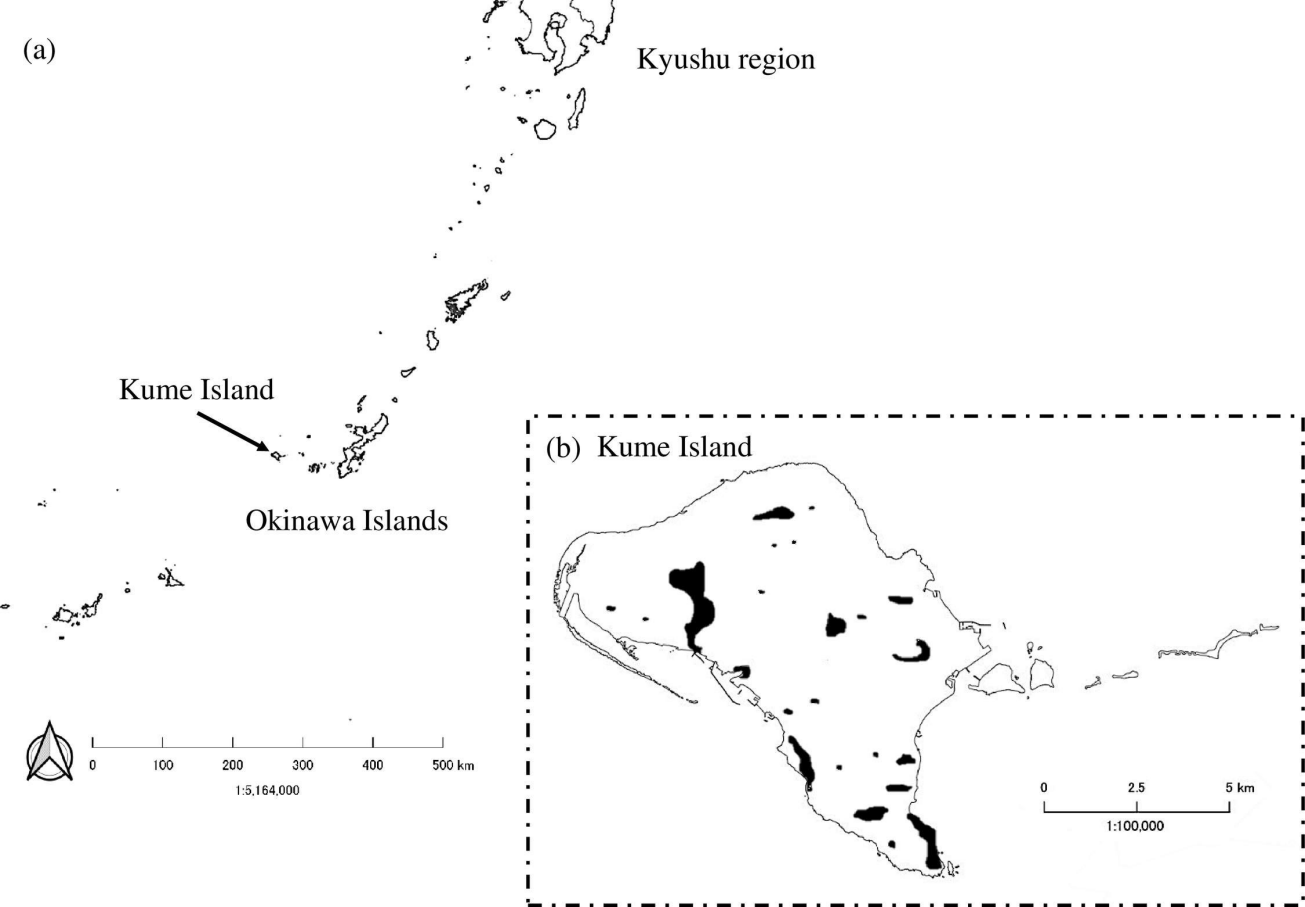
(a) Location of Kume Island, Okinawa, Japan. (b) Predicted distribution area of *C*. *formicarius* in Kume island (areas where male weevils have been found in trap surveys are blacked out). Created by processing a base map (Geospatial Information Authority of Japan).

Kume Island (63.65 km^2^) is located approximately 100 km west of Okinawa Main Island, Japan (26°20N, 126°48E; Fig 1A) and is where about 10,000 people live. The western, eastern, and south-central parts of the island are cultivated with sugarcane, sweet potatoes, and vegetables, while the northern and southeastern mountains are covered with wild vegetation, mainly comprised of subtropical broad-leaved forests and pine forests. The wild host of *C*. *formicarius*, blue morning glory, *Ipomoea indica* (Burm.) Merr., is typically found in the fringe of forests or abandoned lands on the island (Fig 1B). Another major wild host plant of the weevil, railroad vine, *Ipomoea pes-caprae* (L.) Sweet, is widely distributed on sandy seashores. *Cylas formicarius* first invaded the island in 1903 [18].

### Distribution survey and population estimation as baseline data

The surveys of *C*. *formicarius* distribution were conducted from April 1994 to July 1995. We used a sticky board trap (200 mm in height, 100 mm in width; Earth Chemical Co., Tokyo, Japan) with a rubber dispenser containing 0.1 mg of a synthetic sex pheromone, (Z)-3-dodecen-l-ol (E)-2-butenoate (synthesized by Shin-Etsu Chemical Co., Tokyo, Japan) [27] to attract male weevils. The traps mounted on glass fiber sticks were set at a height of 30 cm above the ground and were placed 80–120 m apart along the roads, except for the forest areas and the coastlines where access was difficult. The total number of traps was approximately 3000. Traps were inspected a day or two later, and the number of males captured was counted in the laboratory. The survey was conducted once per site. The population density of *C*. *formicarius* on Kume Island had been previously estimated using mark-release-recapture methods [28].

### Density suppression using MAT application

Since females of *C*. *formicarius* use sex pheromones to attract males, traps with synthetic pheromones can be used to monitor populations. They can also be used to selectively attract and kill males (male annihilation technique (MAT) [27, 29, 30]. Consequently, female reproduction is prevented and the population density decline. Thus, the MAT was used to reduce the male population and consequently the population density before SIT application.

The suppression of weevils by MAT application was conducted from November 1994 to January 1999 in the area north of the center of the island where the density of weevils was high. Fiberboard blocks (45 × 45 × 9 mm or 60 × 60 × 9 mm) containing 0.1 mg of a synthetic sex pheromone [27] and 500 mg of fenitrothion (Sankei Chemical Co., Kagoshima, Japan [31]) were used for the MAT. The fiberboard blocks were applied at a density of 8 per hectare per month in forest areas and cultivated fields (approximately 800 ha), and at 16 per hectare per month in residential areas (approximately 200 ha). Two methods of distribution were used: (1) aerial application by helicopter for forests and cultivated fields and (2) manual ground application for residential areas. Approximately 400000 fiberboard blocks (approximately 240000 by helicopter and approx. 160000 manually) were distributed during MAT application.

### Eradication with SIT application

#### Mass-rearing

We mass-reared two different strains separated by time periods. The reasons for using different strains are detailed in section ‘Marking’ and the Discussion.

**Fig 2:**
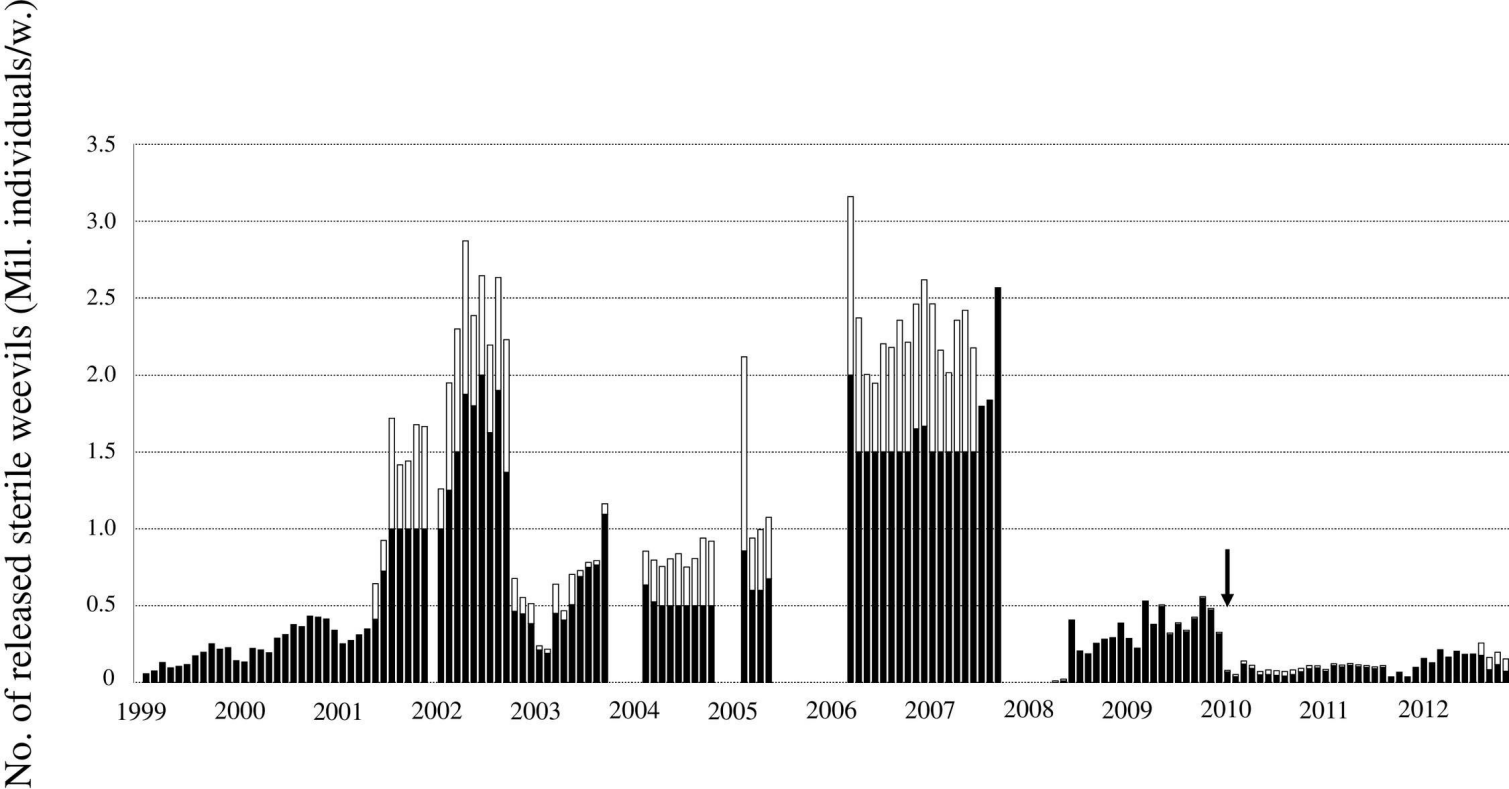
Number of sterile weevils *C*. *formicarius* released per week on Kume Island from 1999 to 2012. Black bars represent aerial releases and white bars represent ground releases. Short breaks in the release of sterile insects were implemented (January-February 2002, January-March 2004, January-March 2005, August 2005-March 2006, December 2007-March 2008) to allow for the detection of remaining areas of occurrence of *C*. *formicarius* using sex pheromone traps. The black arrow indicates the initiation of the release of the PE strain for SIT application in January 2010. Until July 2001, only males were released. However, thereafter, both sexes were released.

*1997~2010*: *Wild strain*. The mass-reared *C*. *formicarius* wild strain (WS) (with bluish or greenish elytra) used initially for SIT application originated from adults collected at Yomitan Village, Okinawa Main Island, Japan (26°24’N, 127°43’E) in October 1997. Weevils were reared on sweet potato roots at 25 ± 1°C and a photoperiod of L14:D10 (light between 04:00 and 18:00) at the Okinawa Prefectural Plant Protection Center (OPPPC) in Naha, Okinawa. The founder population comprised of approximately 10000 individuals. Approximately 2000 weevils were placed per plastic container (290 × 360 × 120 mm) with a mesh lid and approximately 1800 g of sweet potato roots for egg-laying substrate and food. The sweet potatoes were replaced with fresh roots twice a week. The roots with eggs laid on them were stored under the same laboratory conditions as described above. Six and seven weeks after inoculation with weevils, newly emerged adult weevils were collected. Each week, 200000–300000 individuals were used for progressive rearing, and the remainder were sterilized and released. Release of sterile insects started in February 1999. Fig 2 depicts the total number of sterile weevils released per week.

*2008~2012*: *Piceous elytra strain*. The piceous elytra (PE) strain used for SIT application originated from one adult female and one adult male collected at Yonaguni Island, Okinawa, Japan (24°28’N, 123°0’E) in July 2007 and one adult female from a mass-reared wild strain (WS) at OPPPC. The PE was found at a very low frequency in the mass-rearing WS strain. All three showed a piceous elytra. The three weevils were placed in a plastic container (90 in diameter, 20 mm in height) with a mesh lid and approximately 50 g of sweet potato roots for egg-laying substrate and food. The sweet potatoes were replaced with fresh roots on a biweekly basis. The roots with eggs laid on them were stored under the same conditions as described above. Only piceous elytra weevils were visually selected from the newly emerged individuals, and they were allowed to mate and lay eggs. After the 5th generation, no bluish or greenish elytra individuals were found. From the 6th to the 10th generation, 200 adults and two sweet potato roots (approx. 1000 g) were placed in a plastic container (216 × 309 × 238 mm) with a mesh lid. The basic methods of mass-rearing after the 10th generation were the same as those described above for the WS strain.

#### Sterilization

Adult weevils were irradiated with gamma rays from Co^60^ for sterilization. The irradiation dose for sterilization was determined to be 100 or 200-Gy. Since most of the weevils remained in the sweet potato roots for ca. 10-days after adult emergence [12], we used 13- to 16-day-old sexually mature adult weevils. The irradiation facility of the OPPPC was designed by the Radiation Application Development Association, Takasaki, Gunma, Japan. There is a trade-off between insect quality and fertility, as higher radiation doses have a small negative effect on the insect quality, but lower doses do not result in complete sterility [32]. Therefore, insects planned to be made incompletely sterile received lower radiation doses (100-Gy) in the early stage of the eradication program, and insects to be made completely sterile received higher radiation doses (200-Gy) in the late stage of the eradication program. This is because incompletely sterile insects can be more effective in the early stage of control when the wild pest population density is still relatively high. Mathematical models have been developed to determine the switching time between the release of incompletely and completely sterile insects [33, 34]. In *C*. *formicarius*, a fully sterilizing dose was above 200-Gy, slight fertility, and higher survival rates were observed at doses of 100-Gy. At doses of 300-Gy and above, male sexual competitiveness and survival rate were reduced compared to the 200-Gy dose [35]. Thus, a sterilization dose of 100-Gy was used in the early stage of the eradication program (February 1999 to September 2000). From October 2000, we changed the irradiation dose to 200-Gy. The decision was also based on field studies that showed that males irradiated with a 200-Gy dose at the adult stage had a dispersal ability equal to that of non-irradiated males [36]; this dose also had no major effects on male mating behavior for approximately a week after irradiation [37].

#### Marking and selection of morphological marker

In the early stage of the eradication program, sterile weevils were marked with a fluorescent powder dye (0.1 g/10000 individuals) (Braze Orange; DayGlo Color Corp., Cleveland, OH, USA) to distinguish them from wild weevils when captured from the field using monitoring traps baited with pheromone lures. However, the fluorescent powder dye can be lost from the marked weevils and contaminate wild weevils inside the trap, thereby making it difficult to discriminate between the marked-and-released weevils and the “unmarked” wild weevils [38–40]. This was consistent with the fact that the number of marked and unmarked wild weevils trapped appeared to be in sync during the period when the WS strain was used for the SIT releases (see Results and S1 Fig).

With regards to insect marking methods, visible morphological variations, such as body and eye color, have often been used as markers to identify insects [41]. *Cylas formicarius* has three elytral color polymorphisms: bluish, greenish, and piceous elytra (PE) [15, 42–44]. While bluish and greenish elytra are the major color morphs (common morphs) in *C*. *formicarius*, the PE morph is found at a very low frequency (approximately 1%) in some islands of Japan. The scarcity of the PE morph in the field is an advantageous factor when used as phenotypic maker for visible marking of sterile *C*. *formicarius* weevils during SIT application [39, 45]. Therefore, from January 2010 to the later stage of this eradication program, the PE strain was used as a visible marker to mitigate any contamination or detachment of the powder markings. The mating performance, survival, and dispersal ability of the PE males that were mass-reared in OPPPC did not differ from those of WS mass-reared males [39, 40].

#### Sterile insect release

Aerial releases of marked sterile adult weevils were initiated in February 1999. Approximately 1000–3000 weevils (13- to 16-day-old) were dispensed per paper bag (90 × 200 mm) with 4–8 g of vermiculite as a weight material absorbent. These bags were dropped from a helicopter once a week over the designated habitat areas of *C*. *formicarius*. The top of each bag was torn off just before dropping from the helicopter, allowing the weevils to escape once the bags reached the ground. The number of released sterile weevils varied from several hundred to 30000 per hectare depending on the progress of the control measures, density of the wild weevil population, and size of the treatment area. Basically, irradiation, marking, and releasing were carried out in one day.

From June 2001, we initiated the ground release of *C*. *formicarius* by hand into high-density areas, in addition to their aerial release. Approximately 1000–3000 weevils were placed into meshed plastic cups (200 ml) with 4–8 g of vermiculite. We released 500000–3000000 weevils per week by ground. The number of weevils released during the all period is shown in Fig 2.

Until July 2001, only males were selected for release using sex pheromones and used as sterile insects [46]. However, thereafter, both sexes were used.

Since drop-off and contamination of markings on wild weevils was suspected (section ‘Marking’), breaks in the release of the sterile insects were implemented for short periods of time (January-February 2002, January-March 2004, January-March 2005, August 2005-March 2006, December 2007-March 2008) (Fig 2) to identify the remaining areas of occurrence of the wild *C*. *formicarius* population using sex pheromone traps.

#### Evaluation of pest control effectiveness

**Fig 3:**
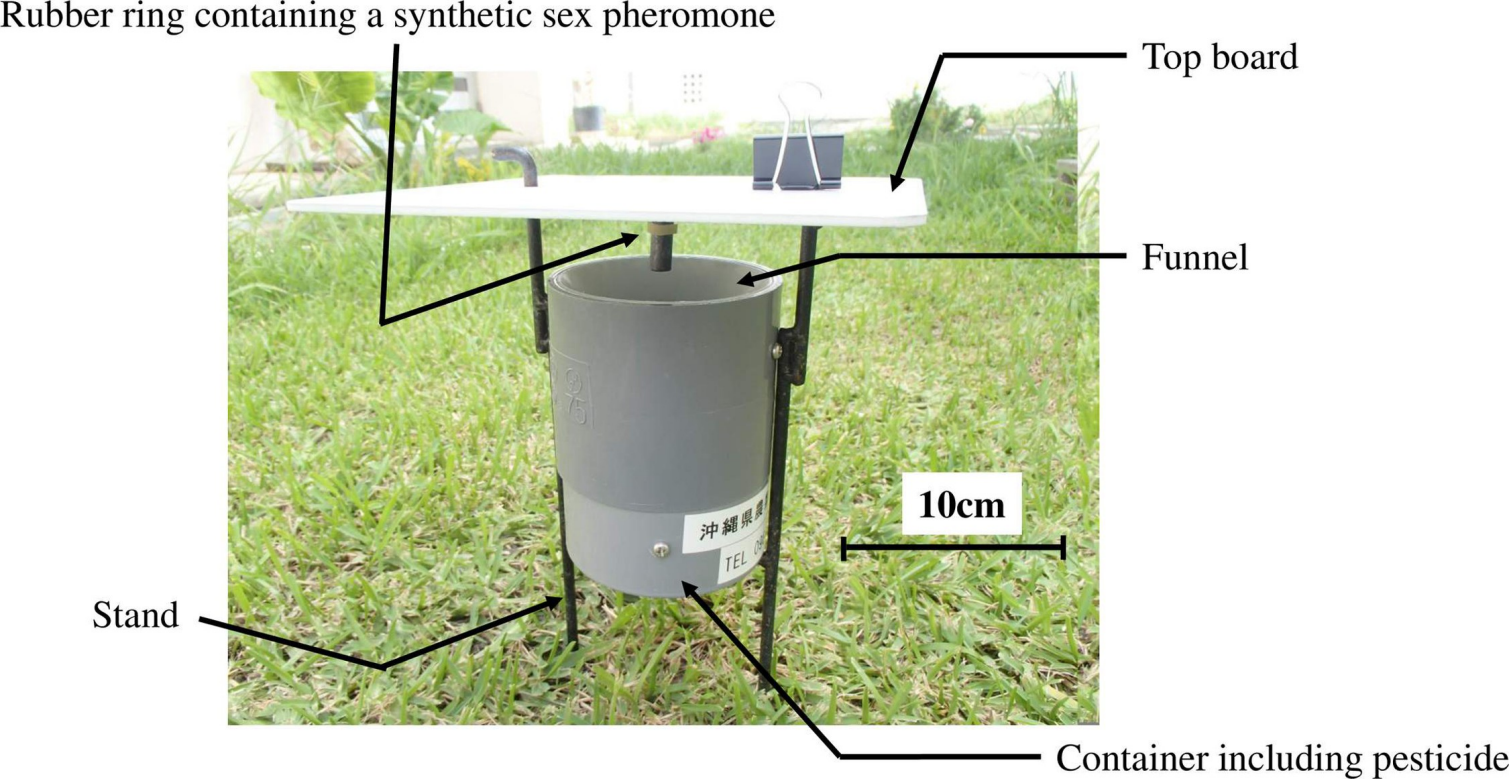
Cylinder-type sex pheromone trap used after April 1998. Traps were fitted with a rubber septum or ring containing 0.1 mg of a synthetic pheromone as an attractant. Insecticide (DDVP) was added to prevent the escape of the trapped insects.

Both sex pheromone traps and host plant surveys were used to evaluate the control efficacy of MAT and SIT application. Two types of funnel sex pheromone traps, cone-type [47] and cylinder-type (Fig 3, [48, 49]), were used to monitor weevil populations. Cone-type traps were only used during the early stage of MAT application. On the other hand, cylinder-type traps were mainly used after April 1998 and during the SIT application period. The efficiency of catching male weevils in cylinder-type traps is the same as in cone-type traps, but it is difficult for other insects to enter the traps [48], so we changed the traps to improve the efficiency of our work.

*Sex pheromone trap*. The traps were fitted with a rubber septum or ring containing 0.1 mg of a synthetic pheromone (Shin-Etsu Chemical Co., Tokyo, Japan) as an attractant, and insecticide (Panaplate^®^, vinyl chloride resin containing 16% DDVP) was added to prevent the escape of trapped insects. 60 to 80 traps were set up in all areas except the forest, and they were inspected twice a month. Under MAT application from 1994 to 1999, the number of traps in MAT application areas was 33, while the number of traps in non-MAT application areas rangeed from 27 to 47. Traps were serviced by replacing the rubbers and insecticides once a month.

*Inspection of host plant infection*. ***Wild hosts*: *Blue morning glory Ipomoea indica and railroad vine Ipomoea pes-caprae*.** To monitor the infestation rates of the weevils, the two wild host species were collected regularly. From May 1995 to July 2000, sclerotized vines were collected once every 1–2 months at 5–15 sites each time, and the length of the collected vines reached from 400 to 1500 m per month. After August 2000, the vines were collected 1–4 times per month from over 20 sites per month (>100 sites in several months). The length of the collected vines reached 2500 to 20000 m per month.

The collected vines were immediately transported to the OPPPC by air. The stems were cut into 1-m sections and split to search for the weevils. The number of larvae, pupae, and adults was counted, and the infection rate per meter of stem was calculated.

*Agricultural products*: *Sweet potato Ipomoea batatas*. A total of 200 plants (including stems over 50 cm from the base and all tubers) were collected from 20 commercial cultivation fields, 10 plants per field, four times a year (every three months). The collected tubers were weighed, counted, and transported to the OPPPC by air. The stems of the collected plants were split to search for weevils. The number of larvae, pupae, and adults was counted, and the infection rate per stem was calculated. The tubers were stored at 25°C for 50 days and then dissected to inspect for adults and pupae.

Because the data corresponding to the number of tubers from 1998 to 2000 were lost, we estimated the number from the total weight of tubers collected, assuming that each had an average weight of 200 g per tuber, based on our other data.

### Statistical analysis

The method proposed by Kuno [50, 51] was used to evaluate the eradication. The relationship between the required number of consecutive zero host plants sampling units, *n*_*0*_ (1 m of stem or a piece of sweet potato tuber), and the marginal infection rate, *p*_*0*_ *(*so low it was regarded as virtually zero), with the error probability,α, was expressed as

$$n_{0}=\frac{\text{log}\alpha }{\text{log}\left(1-p_{0}\right)}$$

With *p*_*0*_ = 0.0001 and *α* = 0.01, the required number of sampling units, *n*_*0*_, was 46049. Therefore, we considered wild weevils to be eradicated when infection by the weevil was not detected continuously in 46050 sample units.

Cross-correlation function (CCF) of the number of marked and unmarked weevils captured by traps per month (S1A and S1B Fig) was calculated by JMP ver. 14.2.0 (SAS Institute Inc., Cary, NC, USA). Autocorrelation coefficient (ACC) and partial autocorrelation coefficient (PACC) of the number of marked and unmarked weevils captured by traps per month (S1C and S1D Fig) were also calculated by JMP ver. 14.2.0.

## Results

### Estimation of pre-project distribution and density of weevils

**Fig 4:**
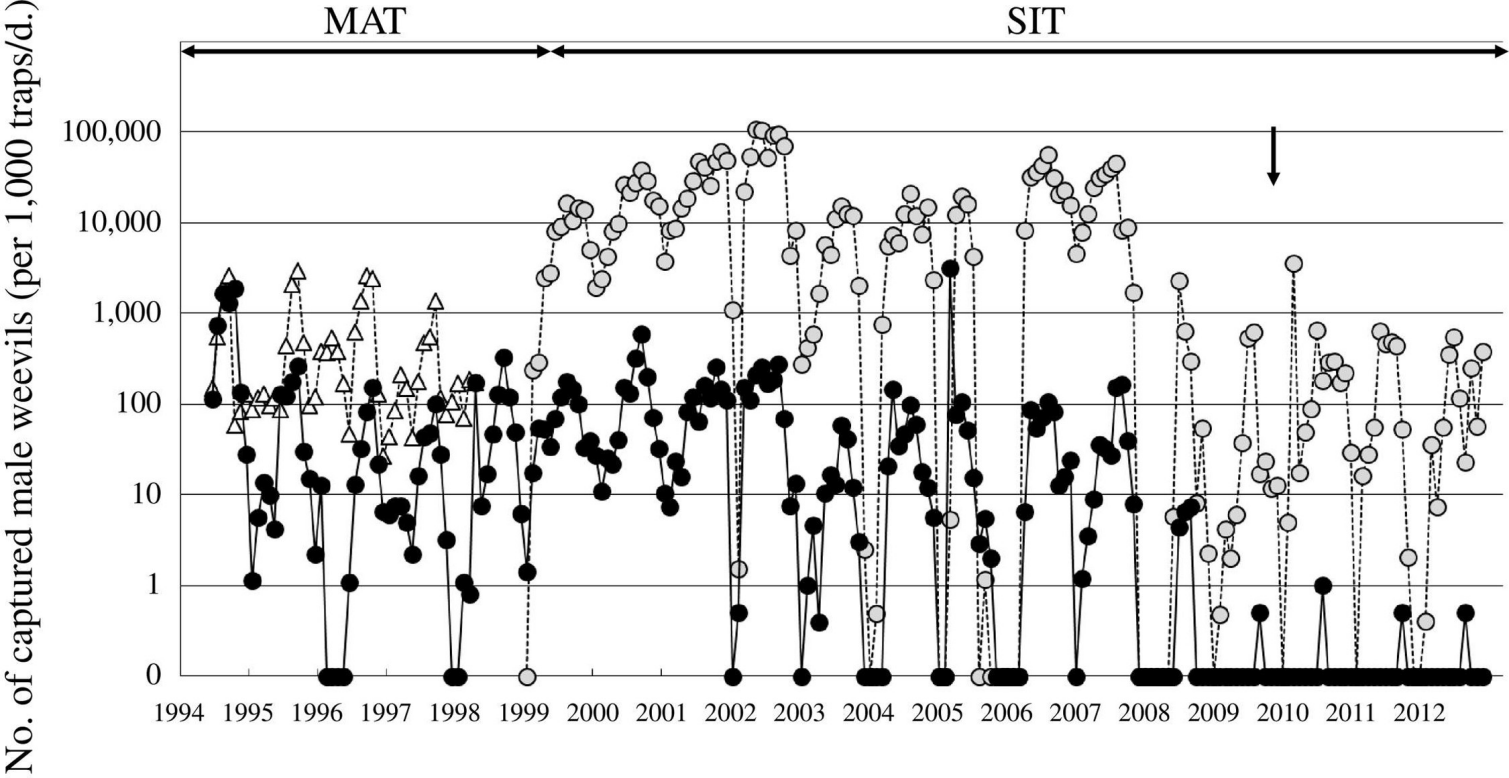
Number of captured male weevils *C*. *formicarius* per thousand traps per day in Kume Island. Black circles indicate wild weevils (unmarked) in the MAT and SIT application area. White triangles indicate wild weevils in the non-MAT application areas. Gray circles indicate marked weevils (from January 2010, we used the phenotypic marker; the piceous elytra of the PE strain) in the SIT application area. The black arrow indicates the initiation of the release of the PE strain for SIT application in January 2010.

The pre-project distribution of *C*. *formicarius* on Kume Island is shown in Fig 1B. Host plant species, especially *I*. *indica*, were found almost everywhere on the island, however, the distribution of weevils was restricted to relatively small areas; they were more abundant in southeastern areas. The number of males caught by traps peaked between August and October and between February and April, and very few were caught during the winter months (December to January) (Fig 4). The highest number was in September 1994, when approximately 2300 weevils were caught per thousand traps per day. The number of wild male weevils during the peak season (September to October) of 1994 was estimated by mark-release-recapture methods to be half a million [28].

### Density suppression using MAT application

**Fig 5:**
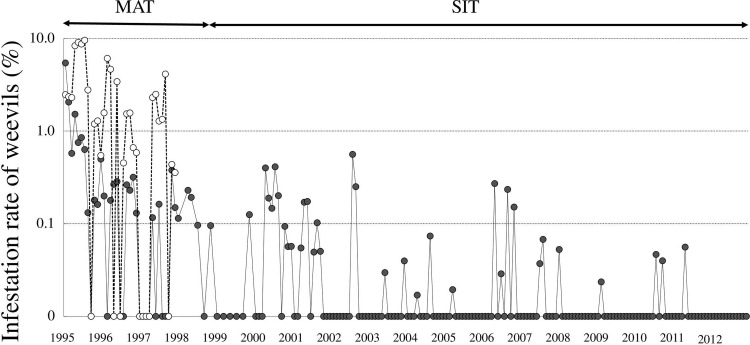
Infestation rate of weevils *C*. *formicarius* on wild hosts in Kume Island. Black circles indicate the % infection in MAT and SIT application areas and white circles indicate % infection in the non-MAT application areas.

The effects of MAT application are shown in Figs 4 and 5. During MAT suppression, the number of weevils caught per thousand traps per day ranged from 83 to 332 at the highest levels. The adult population had decreased by more than 86% in September 1998 compared to September 1994, that is, before the implementation of the MAT (Fig 4). Before this period, the infestation rate of weevils on wild hosts was 9.5%, which decreased gradually to 0.09% in 1998 after the implementation of MAT application (Fig 5).

### Pest control with SIT application

#### Evaluation of pest control effectiveness

*Sex pheromone trap*. During SIT application, the number of weevils attracted to the traps varied from several dozen to several hundred between 1999 and 2009, with no clear downward trend (Fig 4). Statistical synchrony was found while analyzing the dynamics of the capture of marked and unmarked weevils (cross-correlation analysis, CCF = 0.6240 was highest when lag = 0; *p*<0.05) (S1A Fig) from February 1999 to December 2009. After the release of sterile weevils was switched from the WS to the PE strain (January 2010 to December 2012), the statistical synchrony disappeared (S1B Fig) and wild weevils were caught only four times in four years.

*Inspection of host plant parasitism*. **Wild hosts: Blue morning glory *Ipomoea indica* and railroad vine *Ipomoea pes-caprae*.** As shown in Fig 5, no infection was found from March to December 1999. In contrast, after intensive surveys from August 2000 to December 2001 (collecting 4500–20000 meters of vines per month), seven sites of infection were found. As a result of additional releases of sterile insects at these sites, the infection rate returned to zero from January to October 2002, including in the peak season. The infection rate became 0% gradually from 2002 to 2011. No weevils were found in October 2011. Thereafter, 88333 samples from 580 sites were examined, and the infection rate constantly remained zero (>46050 samples; [50, 51]) since October 2011. As a result, weevils were considered eradicated on December 28, 2012.

*Agricultural products*: *Sweet potato Ipomoea batatas*. Between 1995 and 1996, infections in the stems and tubers of sweet potato were detected in two and five cases, respectively. However, from November 1996 to December 2012, no weevils were detected in the stems or tubers **of cultivated sweet potatoes**, despite examining 12748 stems and 48749 tubers. This indicates that the weevils were eradicated (>46050 samples; [50, 51]).

## Discussion

This is the first report of a successful eradication of a Coleoptera population over a wide area using a combination of MAT (male annihilation technique) and SIT (sterile insect technique) application. The eradication project lasted 19 years from its commencement with a pre-project phase to its successful completion, involving the inspection of 1400 km of wild host plants and over 10 tons of sweet potatoes, as well as 6.5 million adult male weevils entrapped using the pheromone trap. Moreover, ca. 460 million sterile weevils were released during the SIT application. The total cost of the project was 4.5 billion yen (equivalent to 56 million USD in 2012). A similar successful project to eradicate the melon fly *B*. *cucurbitae* using SIT application was also undertaken on Kume Island; their absence lasted six years and involved the release of approximately 280 million sterile flies [52]. In comparison to the melon fly, the eradication of weevils took longer due to differences in the ecological characteristics of the insect species, including differences in the dispersal ability of sterile insects of the target species, which may have affected the effectiveness of the SIT [53, 54]. The world’s second eradication of *C*. *formicarius* in 2020 on the island of Tsuken (approximately 1.88 km^2^) in Okinawa took 13 years [55].

The pre-project surveys showed that, although the host plants were distributed throughout the island, the distribution of weevils was restricted to relatively small areas (Fig 1). The number of males caught by traps was particularly high in the southeast area of the island, which was in contrast to lower populations in other areas. The dispersal ability of adult females of this species is known to be very low [56, 57]. The marked unevenness in the distribution of this species is likely to be related to the low dispersal ability of the females. The blue morning glory *I*. *indica* was the main plant host of this species, with very few weevils found in properly managed sweet potato fields. One of the reasons for the long duration of the process of weevil eradication was the presence of weevil-infested *I*. *indica* in abandoned paddy fields (wild hosts) located in inaccessible forested areas in the mountains [58].

Since there is a limit to the mass-rearing of sterile insects, one of the keys to successful eradication is effectively suppressing the density of wild pests before the SIT is used [23]. As synthetic pheromones are in practical use in *C*. *formicarius* management [27, 29, 30], the MAT using these pheromones was applied to achieve efficient density suppression. As a result, the *C*. *formicarius* populations were suppressed by approximately 90%, and the plant infestations decreased from 9.5 to less than 0.1% (Figs 4 and 5). The AW-IPM program that integrated the sequential application of the MAT and the SIT was very effective in eradicating *C*. *formicarius* in the field.

Despite the success of the eradication program, several issues were encountered; the three main ones are detailed below.

### Mass-rearing using raw sweet potatoes

Using the raw sweet potato rearing method, it was possible to produce 1–3 million weevils weekly. However, eradication projects in areas larger than Kume Island would require the production of more weevils. Due to seasonal variations in the production and quality of fresh sweet potatoes over the year, the production of weevils is unstable [46] and probably will require the development of an artificial larval diet. In addition, the cost of buying and maintaining raw sweet potatoes is high. To solve these problems and ensure a stable production of *C*. *formicarius*, artificial feed rearing methods are required. Recently an artificial diet for the West Indian sweet potato weevil *Euscepes postfasciatus* (Fairmaire), also a sweet potato pest [59, 60], has been developed [61–63]. Its adaptation to *C*. *formicarius* may provide a solution to this problem.

### Marking

Fluorescent powder dyes have the disadvantage of becoming detached after their application on sterile weevils. Thereafter, these dyes can adhere to wild weevils caught in pheromone traps, hindering the identification of sterile and wild weevils [38–40]. This resulted in the lack of a downward trend in the number of unmarked weevils caught in the traps, even under SIT application, from February 1999 to December 2009 (Fig 4). During this period, a strong positive correlation was observed between the number of captured marked weevils and that of unmarked weevils (S1A Fig). The pattern showing the relationship between the lag and autocorrelation coefficients or partial autocorrelation coefficients was similar for the marked and unmarked weevils (S1C Fig). In addition, unmarked weevils were caught in traps along with the marked weevils after aerial and ground release of sterile weevils, whereas wild weevils were not caught before the release. Statistical analysis and observations suggested that many of the weevils denoted as “unmarked” were not wild weevils; rather they were marked sterile weevils that had lost their dye. The marked (sterile) to unmarked (wild) ratio (S/W) is commonly used as an indicator of the progress being made in suppressing a pest population under SIT application [2, 64]. However, for the reasons given above, the S/W ratio could not be used. One of the reasons for the success of the eradication was the use of the elytra color polymorphism as a morphological marker (PE), which is very rare in the wild. From January 2010 to December 2012, when the PE strain was used for the sterile insect releases, no longer was a synchrony found between the number of captured marked weevils and the number of unmarked weevils (S1B and S1D Fig), indicating the successful prevention of confusion between sterile (marked) and wild (unmarked) weevils. When individuals of the PE strain were crossed with bluish elytra individuals, the following generation was comprised of individuals with greenish elytra. Therefore, great care must be taken to avoid contamination of the mass-rearing colony with wild weevils [40]. In the future, it will be necessary to consider a two-fold identification method using protein marking and other internal markings [41]. However, the best way to evaluate the effectiveness of the SIT releases is by the impact on the host infestation and our data on host infestation shows unequivocally the successful eradication of the pest.

### Degradation of insect quality due to irradiation

The weevils used in the second half of the SIT application period were completely sterile at a dose of 200-Gy, and only maintained the same mating ability as wild weevils for a week [37]. As the amount of absorbed radiation increases, the sterility of insects increases, but both the quality and mating ability of the insects decreases [65]. This is because the gamma rays used to induce sterility have a negative effect on somatic cells, such as those in the midgut epithelium [66]. An alternative approach that allows maintenance of the quality of insects is dose fractionation, which involves the administration of a series of smaller sterilizing doses over time [66]. When the total dose was the same, there was no difference in the degree of sterility between insects subjected to fractionated irradiation and those subjected to single-dose irradiation. Fractionation, however, allows the somatic cell to recover between doses [67, 68] and is time consuming and increases the cost of the process. Kumano et al. [69] developed a fractionated-dose irradiation method in *C*. *formicarius* in which a 200-Gy dose was divided into three 67-Gy doses to achieve complete sterility while maintain normal mating performance for 12 days, which is twice that resulting from a single dose. Using this method, it is expected that the duration of the effect of complete sterility in weevils will double. There remains much room for improvement in conventional sterilization methods using radiation, and the search for a sterilization method that can maintain high insect quality for a long period of time is necessary for promoting efficient SIT methods. The radiation source currently used was CO^60^, which needs to be replaced periodically owing to the short half-life of the source. In addition, handling gamma radiation sources is complex and requires considerable security measures. Therefore, it is desirable to search for more practical alternative sterilization methods, such as high-energy beams or X-rays [see 66].

Additionally, even after eradication, as a countermeasure against re-invasion, maintaining inspection at ports of entry and a system for the early detection and control of the pest is necessary. This can be achieved by performing invasion weevil surveys in combination with public awareness activities. Since the end of the project in 2012, 58 traps have been set up for detection, and no weevils have been caught until July 2021. Weevils have four generations per year [70], meaning that approximately 33 generations have passed without any specimens being detected in the traps. The eradication of the West Indian sweet potato weevil (*E*. *postfasciatus*)—another major agricultural pest affecting sweet potatoes [59, 60]—has been ongoing on Kume Island since 2001, with wild host surveys and sweet potato surveys for this weevil being carried out 1–4 times a month and 4 times a month, respectively, using the same methods as above [71]. Since the eradication of *C*. *formicarius* in December 2012, >780000 wild hosts have been examined by July 2021, and no specimens of this species have been found. Similarly, sweet potato weevils have not been found in sweet potatoes. As of July 2021, Kume Island remains free of *C*. *formicarius*.

## Supporting information

S1 Fig Cross-correlation function (CCF) the number of marked and unmarked weevils captured by traps per month (a) from February 1999 to December 2009, and (b) from January 2010 to December 2012. The horizontal line in the graph indicates the significance level of p = 0.05. At lag 0, which indicates synchrony, the level of significance is exceeded for (a), but not for (b). Autocorrelation coefficient (ACC) and partial autocorrelation coefficient (PACC) of the number of marked and unmarked weevils captured by traps per month (c) from February 1999 to December 2009, and (d) from January 2010 to December 2012.
(PPTX)

## References

[1] Baumhover AH, Graham A J, Bitter BA, Hopkins DE, New WD, Dudley FH. et al. Screw-worm control through release of sterilized flies. J Econ Entomol. 1955; 48: 462–466.

[2] Knipling EF. Possibilities of insect control or eradication through the use of sexually sterile males. J Econ Entomol. 1955; 48: 459–462.

[3] Wyss JH. Screwworm eradication in the Americas. Ann. N. Y. Acad. Sci. 2000; 916: 186–193. doi: 10.1111/j.1749-6632.2000.tb05289.x 11193620

[4] Mastrangelo T, Welch JB. An overview of the components of AW‐IPM campaigns against the New World screwworm. Insects 2012; 3: 930–955. doi: 10.3390/insects3040930 26466720 PMC4553557

[5] Dyck VA, Hendrichs J, Robinson AS. Sterile Insect Technique, Principles and Practice in Area-wide Integrated Pest Management. 2nd ed. Boca Raton, FL, United Sates of America: CRC Press; 2021

[6] Hendrichs J, Vreysen MJB, Enkerlin WR, Cayol JP. Strategic options in using sterile insects for area-wide integrated pest management. In: Dyck VA, Hendrichs J, Robinson AS, editors. Sterile Insect Technique: Principles and Practice in Area-Wide Integrated Pest Management. 2nd ed. Boca Raton, FL, USA: CRC Press; 2021. pp. 841–884.

[7] Klassen W, Curtis CF, Hendrichs J. History of the sterile insect technique. In: Dyck VA, Hendrichs J, Robinson AS, editors. Sterile Insect Technique: Principles and Practice in Area-Wide Integrated Pest Management. 2nd ed. Boca Raton, FL, USA: CRC Press; 2021. pp. 1–44.

[8] Smith JW. Boll weevil eradication: area-wide pest management. Ann Entomol Soc Am. 1998; 91: 239–247.

[9] Hardee DD, Harris FA. Eradicating the boll weevil (Coleoptera: Curculionidae): a clash between a highly successful insect, good scientific achievement, and differing agricultural philosophies. Am Entomol. 2003; 49: 82–97.

[10] Fajardo M., Rodríguez X., Hernández C. D., Barroso L., Morales M., González A., et al. 2021. The eradication of the invasive red palm weevil in the Canary Islands. In: Hendrichs J, Pereira R, Vreysen MJB, editors. Area-Wide Integrated Pest Management: Development and Field Application. Boca Raton, FL, USA: CRC Press; 2021. pp. 539–550.

[11] Horber E. Eradication of the white grub (Melolontha vulgaris F.) by the sterile male technique. In Proceedings, Symposium: Radiation and Radioisotopes Applied to Insects of Agricultural Importance. Athens, Greece: FAO/IAEA; 1963. pp. 313–332.

[12] Sutherland JA. A review of the biology and control of the sweetpotato weevil Cylas formicarius (Fab.). Trop Pest Manage. 1986; 32: 304–315.

[13] Chalfant KL, Jansson RK, Seal DR, Schalk JM. Ecology and management of sweet potato insects. Ann Rev Entomol. 1990; 35: 157–180.

[14] Yasuda K, Kohama T. Distribution of the sweetpotato weevil, Cylas formicarius (Fabricius) and the West Indian sweetpotato weevil, Euscepes postfasciatus (Fairmire) in Okinawa prefecture. Proc Assoc Plant Prot Kyushu, 1990; 36: 123–125.

[15] Wolfe G W. The origin and dispersal of the pest species of Cylas with a key to the pest species groups of the world. In: Jansson RK, Raman KV editors. Sweet Potato Pest Management: A Global Perspective. Boulder: Westview Press; 1991. pp. 13–43.

[16] Akazawa T, Uritani L, Kubota H. Isolation of ipomeamarone and two coumarin derivatives from sweet potato roots injured by the weevil, Cylas formicarius elegantulus. Arc Biochem Biophys. 1960; 88: 150–156. doi: 10.1016/0003-9861(60)90210-1 13792235

[17] Uritani I, Saito T, Honda H, Kim WK. Induction of furano-terpenoids in sweet potato roots by the larval components of the sweet potato weevils. Agr Biol Chem 1975; 39: 1857–1862.

[18] Nawa U. A note of the sweet potato weevil. Konchu Sekai. 1903; 7: 327–330 (in Japanese).

[19] Setokuchi O. Biology and Control of the Sweet potato Weevil, Cylas formicarius Fabricius in the Amami Island. Plant Prot 1990; 44: 111–114 (in Japanese).

[20] Austin DF. Association between the plant family Convolvulaceae and Cylas weevils. In: Jansson R, Raman KKV, editors. Sweet Potato Pest Management, A Global Perspective. Boulder: Westview Press. 1991. pp. 45–57.

[21] Lindquist AW. Biological information needed in the sterile-male method of insect control. In: Proceedings od Panel, Vienne, 27–31 May 1968. Sterile-Male Technique for Eradication or Control of Harmful Insects. Vienna International Atomic Energy Agency 1969. Vienna, Austria: Joint FAO/IAEA Division of Atomic Energy in Food and Agriculture; 1969. pp. 33–37. https://www.iaea.org/sites/default/files/el_harmful_insects.pdf

[22] Lindquist DA, Butt BA, Moore I. (1974). Ecological requirements of the sterile male technique. In Proceedings: FAO Conference on Ecology in Relation to Plant Pest Control, 11–15 December 1972. Rome, Italy: FAO; 1974. pp. 249–262.

[23] Knipling EF. The basic principles of insect population suppression and management. Agriculture Handbook 512, Washington, DC, USA: United States. Department of Agriculture; 1979. 455993

[24] Itô Y, Yamamura K, Manoukis NC. Role of population and behavioural ecology in the sterile insect technique. In: Dyck VA, Hendrichs J, Robinson AS, editors. Sterile Insect Technique: Principles and Practice in Area-Wide Integrated Pest Management. 2nd ed. Boca Raton, FL, USA: CRC Press; 2021. pp. 245–282.

[25] Vreysen MJB, Saleh KM, Ali MY, Abdulla AM, Zhu ZR, Juma KG, et al. Glossina austeni (Diptera: Glossinidae) eradicated on the island of Unguja, Zanzibar, using the sterile insect technique. J Econ Entomol. 2000: 93; 123–135. doi: 10.1603/0022-0493-93.1.123 14658522

[26] Vreysen MJB, Seck MT, Sall B, Mbaye AG, Bassene M, Fall A G. Lo, et al. (2021). Area-wide integrated management of a Glossina palpalis gambiensis population from the Niayes area of Senegal: a review of operational research in support of a phased conditional approach. In: Hendrichs JR, Pereira R, Vreysen MJB, editors. Area-wide integrated pest management. Development and field application. Boca Raton, FL, USA: CRC Press; 2021. pp. 275–303.

[27] Heath RR, Coffelt JA, Sonnet PE, Proshold FI, Dueben B, Tumlinson JH. Identification of sex-pheromone produced by female sweetpotato weevil, Cylas formicarius elegantulus (Summers). J Chem Ecol. 1986: 12; 1489–1503. doi: 10.1007/BF01012367 24307127

[28] Kuba H, Teruya T, Sakakibara M. Eradication of weevils by sterile-insect-release method. (9) Experimental eradication project of sweet potato weevils in Kume Island. Plant Prot, 2000:54; 483–486 (in Japanese).

[29] Jansson RK, Mason LJ, Heath RR. Use of sex pheromone for monitoring and managing *Cylas formicarius*. In: Jansson RK, Raman KV, editors. Sweet Potato Pest Management: A Global Perspective. Boulder: Westview Press; 1991. pp. 97–138.

[30] Yasuda K. Mass trapping of the sweet potato weevil *Cylas formicarius* (Fabricius) (Coleoptera: Brentidae) with a synthetic sex pheromone. Appl Entomol Zool, 1995; 30: 31–36.

[31] Setokuchi O, Nakamura Y, Kubo Y. A fiberboard formulation of the sweetpotato weevil, *Cylas formicarius* (Fabricius), sex pheromone and an insecticide for mass-trapping. Japan Appl Entomol Zool, 1991; 35: 251–253 (in Japanese with English summary).

[32] Robinson AS. Genetic basis of the sterile insect technique. In: Dyck VA, Hendrichs JR, Robinson AS, editors. Sterile Insect Technique: Principles and Practice in Area-wide Integrated Pest Management. 2nd ed. Boca Raton, FL, USA: CRC Press; 2021. pp. 143–162.

[33] Suzuki Y, Miyai S. Eradication of weevils by sterile insect release method (5): making use of partially sterilized insects. Plant Prot, 2000; 54: 469–470 (in Japanese).

[34] Barclay HJ. Modeling incomplete sterility in a sterile release program: interactions with other factors. Popul Ecol, 2001; 43: 197–206.

[35] Kohama T. (1998) Development of technology for sterile insect technique of sweet potato weevil. I Study of adult irradiation. In: Reports of pest and disease test of 1997. Naha, Department of Pests and Diseases, Okinawa Prefectural Agricultural Experiment Station; 1998. pp. 59–60 (in Japanese).

[36] Kumano N, Kohama T, Ohno S. Effect of irradiation on dispersal ability of male sweetpotato weevils (Coleoptera: Brentidae) in the Field. J Econ Entomol, 2007; 100: 730–736. doi: 10.1603/0022-0493(2007)100[730:eoioda]2.0.co;2 17598532

[37] Kumano N, Haraguchi D, Kohama T. Effect of irradiation on mating ability in the male sweetpotato weevil (Coleoptera: Curculionidae). J Econ Entomol, 2008; 101: 1198–1203. doi: 10.1603/0022-0493(2008)101[1198:eoioma]2.0.co;2 18767728

[38] Kuba H, Kohama T, Haraguchi D. (2003). Eradication projects of exotic sweet potato weevils using SIT in Okinawa. In Oka M, Masui M, Shiomi T, Ogawa Y. Tsuchiya K, editors. Proceeding of the NIAWS-FFTC Joint International Seminar on Biological Invasions: Environmental Impacts and the Development of a Database for the Asian-Pacific Region. National Institute for Agro-Environmental Sciences, Tsukuba, and Food and Fertilizer Technology Center for the Asian and Pacific Region, Taipei; 2003. pp. 273–287.

[39] Shiromoto K, Kumano N, Kuriwada T, Haraguchi D. Is elytral color polymorphism in sweetpotato weevil (Coleoptera: Brentidae) a visible marker for sterile insect technique? Comparison of male mating behavior. J Econ Entomol. 2011; 104: 420–424. doi: 10.1603/ec10341 21510188

[40] Shiromoto K, Kawamura K, Haraguchi D, Matsuyama T. Elytral color polymorphism of sweet potato weevil, Cylas formicarius (Coleoptera: Brentidae) as a visible marker for a sterile insect technique in Japan. Plant Prot, 2012; 66: 316–320 (in Japanese).

[41] Hagler JR, Jackson CG. Methods for marking insects: Current techniques and future prospects. Ann Rev Entomol, 2001; 46: 511–543. doi: 10.1146/annurev.ento.46.1.511 11112178

[42] Pierce WD. Weevils which affect Irish Potato, Sweet Potato and Yam. J Agric Res, 1918; 12: 601–611.

[43] Pierce WD. Studies of the sweet potato weevils of the subfamily Cyladinae. Bull S Calif Acad Sci, 1940; 39: 205–223.

[44] Kissinger DG. Curculionidae Subfamily Apioninae of North and Central America. With Reviews of the World Genera of Apioninae and World Genera of Apion Herbst (Coleoptera). South Lancaster, Massachusetts; Taxonomic Publications; 1968.

[45] Kawamura K, Ohno S, Haraguchi D, Kawashima S, Kohama T. Geographic variation of elytral color in the sweetpotato weevil, Cylas formicarius (Fabricius) (Coleoptera: Brentidae), in Japan. Appl Entomol Zool, 2009; 44: 505–513.

[46] Miyaji K, Nishihara S, Hara Y, Tokunaga T, Hatono T, Kamikado T, et al. Eradication of weevils by sterile-insect-release method (6) methods of mass rearing, sterilization, marking, and release of Cylas formicarius. Plant Prot, 2000; 54: 472–475 (in Japanese).

[47] Yasuda K, Sugie H, Heath RR. Field evaluation of synthetic sex-attractant pheromone of the sweet-potato weevil Cylas formicarius Fabricus (Coleoptera: Brentidae). Japan J Appl Entomol Zool, 1992; 36: 81–87 (in Japanese with English summary).

[48] Sugiyama M, Shimoji Y, Kohama T. Effectiveness of a newly designed sex pheromone trap for the sweetpotato weevil, Cylas formicarius (FABRICIUS) (Coleoptera: Brentidae). Appl Entomol Zool, 1996; 31: 547–550.

[49] Kohama T, Toyoguchi T, Sugiyama M, Miyata S. Improved sex pheromone trap for monitoring sweetpotato weevil, *Cylas formicarius* (Fabricius) (Coleoptera: Brentidae). Okinawa Agric, 1999; 34: 30–33 (in Japanese with English summary).

[50] Kuno E. On the assessment of low rate of pest infestation based on successive zero-samples. Japan J Appl Entomol Zool, 1978; 22: 45–46 (in Japanese with English summary).

[51] Kuno E. Verifying zero-infestation in pest control: a simple sequential test based on the succession of zero-samples. Res Popul Ecol, 2001; 33: 29–32.

[52] Iwahashi O. Eradication of the melon fly, *Dacus cucurbitae*, from Kume Is., Okinawa with the sterile insect release method. Res Popul Ecol, 1977; 19: 87–98.

[53] Ikegawa Y, Himuro C. Limited mobility of target pests crucially lowers controllability when sterile insect releases are spatiotemporally biased. J Theor Biol, 2017; 421: 93–100. doi: 10.1016/j.jtbi.2017.03.026 28363862

[54] Lance DR, McInnis DO. Biological basis of the sterile insect technique. In: Dyck VA, Hendrichs J, Robinson AS, editors. Sterile Insect Technique: Principles and Practice in Area-Wide Integrated Pest Management. 2nd ed. Boca Raton, FL, USA: CRC Press; 2021. pp. 113–142.

[55] Plant Protection Station. Plant Protection Information (41) Yokohama Plant Protection Station, Ministry of Agriculture, Forestry and Fisheries; 2021 (in Japanese).

[56] Moriya S. A preliminary study on the flight ability of the sweetpotato weevil, Cylas formicarius (Fabricius) (Coleoptera: Apionidae) using a flight mill. Appl Entomol Zool, 1995; 30: 244–246.

[57] Moriya S, Miyatake T. Eradication of weevils by sterile-insect-release method. (2) Dispersal. Plant Prot, 2000; 54: 459–462 (in Japanese).

[58] Kohama T, Kuba H. Eradication of the Sweet potato weevil by a combination of sex pheromone and sterile insect technique: Current status and problems of control in Kume Island, Okinawa Prefecture. In: Ito Y, editor. Sterile insect technique. Technology for eradicating invasive pests. Tokyo, Japan: Kaiyusha; 2008. pp. 277–316 (in Japanese).

[59] Sherman M, Tamashiro M. The sweetpotato weevils in Hawaii: their biology and control. Hawaii Agric Exp Stn Tech Bull, 1954; 23: 1–36.

[60] Raman KV, Alleyne EH. Biology and management of the West Indian sweet potato weevil, Euscepes postfasciatus. In: Jansson RK, Raman KV, editors. Sweet potato pest management: a global perspective. Boulder: Westview Press, 1991. pp. 263–281.

[61] Shimoji Y, Kohama T. An artificial larval diet for the West Indian sweet potato weevil, Euscepes postfasciatus (FAIRMAIRE) (Coleoptera: Curculionidae). Appl Entomol Zool, 1996; 31: 152–154.

[62] Ohishi T, Honma A, Himuro C, Teruya A. Effect of sweet potato powder content in larval artificial diets on the yield and quality of mass-reared West Indian sweet potato weevil, Euscepes postfasciatus (Coleoptera: Curculionidae). Japan J Appl Entomol Zool, 2018; 62: 123–126 (in Japanese with English summary).

[63] Misa K, Himuro C, Honma A, Ikegawa Y, Ohishi T. Effects of storage periods of an artificial larval diet on the yield and quality of mass-reared West Indian sweet potato weevil (Coleoptera: Curculionidae). J Econ Entomol, 2020; 113: 2613–2618. doi: 10.1093/jee/toaa190 32886105

[64] Knipling EF. Sterile insect technique as a screwworm control measure. The concept and its development. In: Graham OH, editor. Symposium on Eradication of the Screwworm from the United States and Mexico, 62, Misc. Publ. Entomol. Soc. America, 1985. pp. 8–11.

[65] Parker AG, Vreysen MJB, Bouyer J, Calkins CO. Sterile insect quality control/assurance. In: Dyck VA, Hendrichs J, Robinson AS, editors. Sterile Insect Technique: Principles and Practice in Area-Wide Integrated Pest Management. 2nd ed. Boca Raton, FL, USA: CRC Press; 2021. pp. 399–440.

[66] Bakri A, Mehta K, Lance D. Sterilizing insects with ionizing radiation. In: Dyck VA, Hendrichs J, Robinson AS, editors. Sterile Insect Technique: Principles and Practice in Area-Wide Integrated Pest Management. 2nd ed. Boca Raton, FL, USA: CRC Press; 2021. pp. 355–398.

[67] Elkind M, Sutton H. X-ray damage and recovery in mammalian cells in culture. Nature, 1959; 184: 1293. doi: 10.1038/1841293a0 13819951

[68] Ducoff H. Causes of death in irradiated adult insects. Biol Rev, 1972; 47: 211–238. doi: 10.1111/j.1469-185x.1972.tb00973.x 4206386

[69] Kumano N, Kuriwada T, Shiromoto K, Haraguchi D, Kohama T. Prolongation of the effective copulation period by fractionated-dose irradiation in the sweet potato weevil, *Cylas formicarius*. Entomol Exp Appl, 2011; 141: 129–137.

[70] Yasuda K. Studies on integrated pest management of West Indian sweet potato weevil Euscepes postfasciatus (Fairmire) and sweet potato weevil Cylas formicarius (Fabricius). Bull Okinawa agric exp stan. 1998; 21: 1–80.

[71] Okinawa Prefectural Plant Protection Center. Project report on control of special diseases and insect pests in Okinawa Prefecture 2019 (45). Naha: Okinawa Prefectural Plant Protection Center; 2019 (in Japanese).

