# Supplementary material for: First case of successful eradication of the sweet potato weevil, Cylas formicarius (Fabricius), using the sterile insect technique
Source: PLoS One. 2022 May 12;17(5):e0267728. doi: 10.1371/journal.pone.0267728 (PMC9098069; doi:10.1371/journal.pone.0267728)
Supplement: S1 Fig — Cross-correlation function (CCF) the number of marked and unmarked weevils captured by traps per month (a) from February 1999 to December 2009, and (b) from January 2010 to December 2012. The horizontal line in the graph indicates the significance level of p = 0.05. At lag 0, which indicates synchrony, the level of significance is exceeded for (a), but not for (b). Autocorrelation coefficient (ACC) and partial autocorrelation coefficient (PACC) of the number of marked and unmarked weevils captured by traps per month (c) from February 1999 to December 2009, and (d) from January 2010 to December 2012. (PPTX) [file pone.0267728.s001.pptx]

## Slide 1
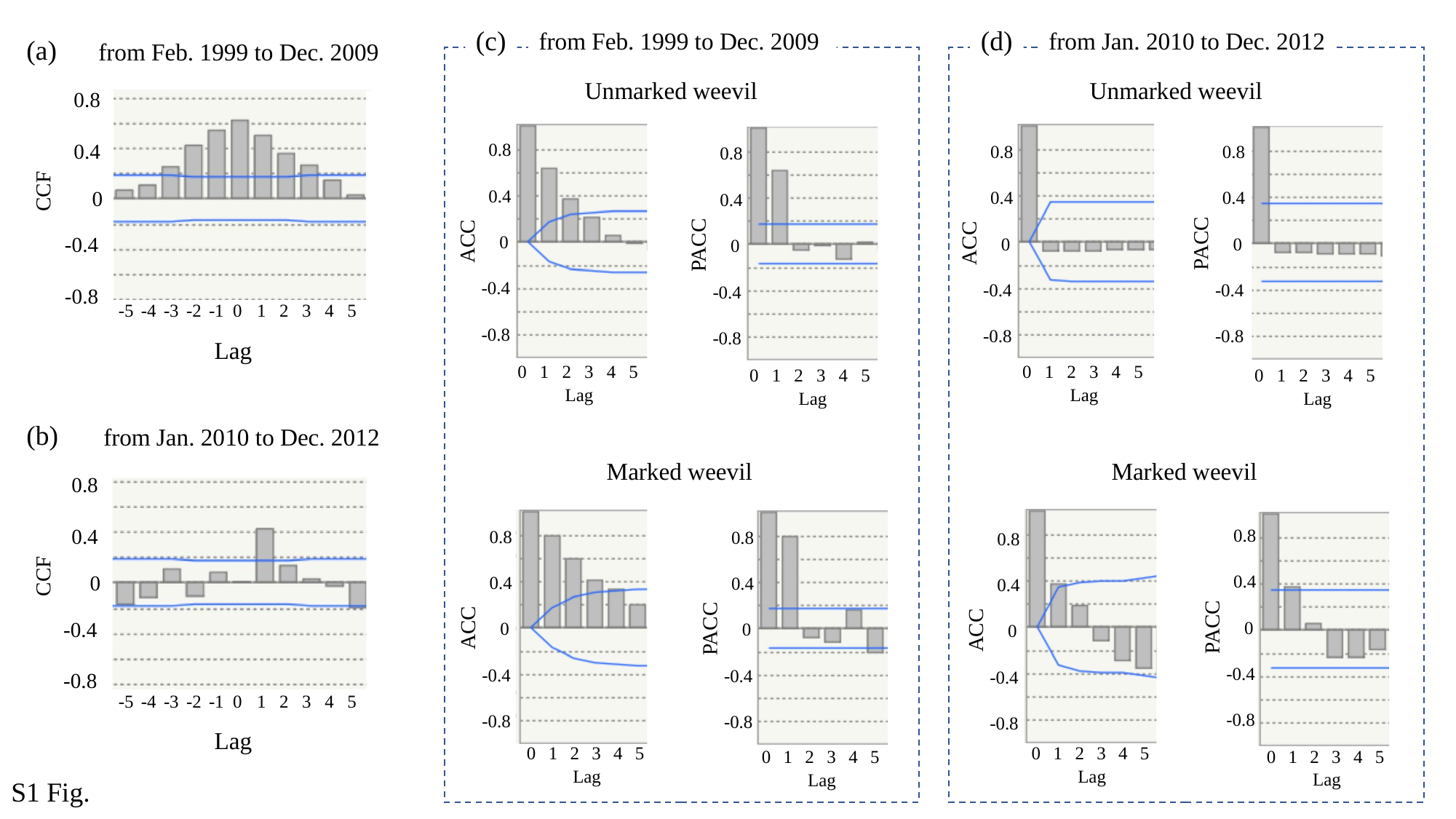

(d)
(c)
from Feb. 1999 to Dec. 2009
Unmarked weevil
0.8
0.4
ACC
0
-0.4
-0.8
0
1
2
3
4
5
0
1
2
3
4
5
Lag
Lag
Marked weevil
0
1
2
3
4
5
0
1
2
3
4
5
Lag
Lag
from Jan. 2010 to Dec. 2012
(a)
from Feb. 1999 to Dec. 2009
Unmarked weevil
0.8
0.4
0.8
0.4
PACC
0
-0.4
-0.8
0.8
0.4
ACC
0
-0.4
-0.8
0.8
0.4
PACC
0
-0.4
-0.8
CCF
0
-0.4
-0.8
-5
-4
-3
-2
-1
0
1
2
3
4
5
Lag
0
1
2
3
4
5
0
1
2
3
4
5
Lag
Lag
(b)
from Jan. 2010 to Dec. 2012
Marked weevil
0.8
0.4
0.8
0.4
PACC
0
-0.4
-0.8
0.8
0.4
ACC
0
-0.4
-0.8
0.8
0.4
PACC
0
-0.4
-0.8
0.8
0.4
ACC
0
-0.4
-0.8
CCF
0
-0.4
-0.8
-5
-4
-3
-2
-1
0
1
2
3
4
5
Lag
0
1
2
3
4
5
0
1
2
3
4
5
Lag
Lag
S1 Fig.
